# Neurotoxicity Associated with Treatment of Acute Lymphoblastic Leukemia Chemotherapy and Immunotherapy

**DOI:** 10.3390/ijms23105515

**Published:** 2022-05-15

**Authors:** Patrycja Śliwa-Tytko, Agnieszka Kaczmarska, Monika Lejman, Joanna Zawitkowska

**Affiliations:** 1 Student’s Scientific Association at the Department of Pediatric Hematology, Oncology and Transplantation, Medical University of Lublin, A. Racławickie 1, 20-059 Lublin, Poland; sliwa.pat@gmail.com; 2 Student Scientific Society, Laboratory of Genetic Diagnostics, Medical University of Lublin, A. Racławickie 1, 20-059 Lublin, Poland; agnieszkakacz70@gmail.com; 3 Laboratory of Genetic Diagnostics, Medical University of Lublin, A. Racławickie 1, 20-059 Lublin, Poland; monika.lejman@umlub.pl or; 4 Department of Pediatric Hematology, Oncology and Transplantology, Medical University of Lublin, A. Racławickie 1, 20-059 Lublin, Poland

**Keywords:** acute lymphoblastic leukemia, neurotoxicity, immunotherapy, chemotherapy

## Abstract

Immunotherapy is a milestone in the treatment of poor-prognosis pediatric acute lymphoblastic leukemia (ALL) and is expected to improve treatment outcomes and reduce doses of conventional chemotherapy without compromising the effectiveness of the therapy. However, both chemotherapy and immunotherapy cause side effects, including neurological ones. Acute neurological complications occur in 3.6–11% of children treated for ALL. The most neurotoxical chemotherapeutics are L-asparaginase (L-ASP), methotrexate (MTX), vincristine (VCR), and nelarabine (Ara-G). Neurotoxicity associated with methotrexate (MTX-NT) occurs in 3–7% of children treated for ALL and is characterized by seizures, stroke-like symptoms, speech disturbances, and encephalopathy. Recent studies indicate that specific polymorphisms in genes related to neurogenesis may have a predisposition to MTX toxicity. One of the most common complications associated with CAR T-cell therapy is immune effector cell-associated neurotoxicity syndrome (ICANS). Mechanisms of neurotoxicity in CAR T-cell therapy are still unknown and may be due to disruption of the blood–brain barrier and the effects of elevated cytokine levels on the central nervous system (CNS). In this review, we present an analysis of the current knowledge on the mechanisms of neurotoxicity of standard chemotherapy and the targeted therapy in children with ALL.

## 1. Introduction

Five-year overall survival of ALL has increased over the past decades and now exceeds over 96% [1]. Chemotherapy is a crucial part of treating ALL and involves many cytotoxic drugs, which inhibit cancer cells from growing rapidly, but they also damage healthy cells, resulting in a wide range of adverse effects. However, with the current high rate of survival, it would be difficult to improve results with only conventional chemotherapy which has reached its maximum of tolerance and could no longer be pushed to improved results. The target should be to search for the use of intensive multimodal treatment regimens, including high-dose chemotherapy and next-generation drugs [2]. Precision medicine with immunotherapy and other molecularly targeted treatments offers unique opportunities to customize treatment intensity [3]. Their advantages also include reducing the need for hematopoietic stem cell transplantation (HSCT), decreasing the burden of toxicities, and fighting persistent residual disease. Recently approved agents for ALL include blinatumomab, inotuzumab ozogamicin (InO), and CAR T-cell therapy, which are expected to improve treatment outcomes and reduce doses of conventional chemotherapy without compromising the effectiveness of the therapy. Nevertheless, the benefits of aggressive chemotherapy versus target therapy for different patient groups remain unclear and all the strategies cause adverse events, such as neurotoxicity, hepatotoxicity, gastrointestinal complication, and secondary malignancies, making neither of these therapies an ideal treatment [4].

Acute neurological complications occur in 3.6–11% of children treated for ALL. Some cytostatic agents (L-ASP, MT, VCR, Ara-G) that are an essential component of modern ALL therapy can cause neurological problems during treatment [5]. Moreover, it is believed that successive advances in immunotherapy, especially CAR T-cell therapy, may be the next generation of treatment for ALL. Despite the potential of CAR T-cell therapy, it is associated with a variety of side effects, including cytokine release syndrome (CRS) and ICANS. Both clinical symptoms can be mild to life threatening in intensity [6].

In this review, we present an analysis of the current knowledge on the complications and mechanisms of neurotoxicity of standard chemotherapy and the targeted therapy in children with ALL and provide information on their acute neurological outcomes.

## 2. Neurotoxicity of Conventional Therapy

The optimal treatment doses are determined based on tolerability, response assessment, and drug pharmacodynamics and pharmacogenomics. The clinical characteristics of the patient and the biological features of the leukemia are the main factors that determine the choice of specific chemotherapeutics. Treatment protocol consists of phases such as induction, intensification, consolidation, and maintenance [7]. Currently, first-line treatment protocols include a variety of medication combinations which involve the use of VCR, L-ASP, corticosteroids, antimetabolites (cytarabine and MTX), and anthracyclines [3]. However, the dose intensity of conventional chemotherapy has been pushed to its limits, and because children absorb and metabolize drugs differently than adults, toxicity is a key issue in pediatric chemotherapy. To determine further treatment development, attention must be given to some of the unique neurotoxicities associated with MTX, VCR, L-ASP, and their molecular background (Table 1).

### 2.1. Methotrexate

Methotrexate is an essential drug in the treatment of ALL in children. MTX can be administered orally, intravenously (I.V.), or intrathecally. When given intrathecally, it prevents leukemic cells from entering the cerebrospinal fluid (CSF) around the spine and brain. MTX is critical in the systemic control of leukemia and the prevention and treatment of critical locations including the CNS. Methotrexate associated with neurotoxicity (MTX-NT) occurs in 3–7% of treated children for ALL [21]. Despite many studies, it is still unknown what causes MTX-NT. MTX neurotoxicity is defined as symptomatic neurotoxicity, with or without leukoencephalopathy, that is temporarily linked to intravenous or intrathecal MTX injection and has been ruled out by other causes. Moreover, there are many drug–drug interactions that potentially increase MTX neurotoxicity or affect the efficacy of systemic chemotherapy, such as anti-epileptic medications, the risk of which during therapy should be systematically evaluated. Initially, the etiology of MTX-NT was thought to be primarily related to brain folate homeostasis, but the precise mechanism is unclear and may be multifunctional. Nevertheless, some mechanisms seem most likely to be involved. MTX and its metabolites are known to inhibit 5-aminoimidazole-4-carboxamide ribonucleotide formyltransferase (ATIC) and promote adenosine release. Adenosine binds with the adenosine receptor and modifies neuronal response in the CNS [22].

It is known that in the metabolic pathway of any drug, there are many interactions between genes and their products that lead to proper functioning. MTX is absorbed into the cell by active transport. The membrane protein encoded by the Solute Carrier Family 19 Member 1 (*SLC19A1*) gene is a folate transporter and is involved in the regulation of intracellular folate concentration. A mutation in *SLC19A1* (c.80G > A) leads to the conversion of the amino acid arginine to histidine at codon 27 (Arg27His), located in the first transmembrane domain near the binding site to the outer plasma membrane [23]. Previous studies suggest that this change likely modifies the structure of the transporter and affects its function [24]. Recently, studies have shown the association of adenosine and its receptor (*ADORA1*, Adenosine A2A Receptor [*ADORA2A*]) with many CNS disorders such as attention deficit hyperactivity disorder (ADHD) and Gilles de la Tourette syndrome [25]. *ADORA2A* activation in the CNS promotes neuronal damage by enhancing excitatory neurotransmitters and can also induce white matter damage. Two of the *ADORA2A* polymorphisms, rs2298383 and rs5760410, are associated with neurotoxicity. It has been shown that patients with rheumatoid arthritis treated with MTX having the rs2298383 and rs5760410 polymorphisms are associated with a higher incidence of adverse events. The *ADORA2* polymorphism rs2298383 has three genotypes: CC, CT, and TT. However, the treatment-related serious adverse events have a major impact on overall survival, and they are manifested as seizures, aphasia, mental status disorder, stroke-like episodes, delirium, or even leukoencephalopathy [26].

Tsujimoto et al. reported the study, which included 56 patients. The study aimed to check whether *ADORA* polymorphisms also affect the risk of leukoencephalopathy in children with hematologic malignancies. Out of 56 patients, 21 (37%) developed leukoencephalopathy during treatment. Neurotoxic side effects occurred in three of four patients who received triple intrathecal therapy (TITs) and in one patient after intermediate-dose (500 mg/m^2^/day) methotrexate (ID-MTX). Two patients developed stroke-like symptoms, one patient had delirium, and one patient experienced a seizure. MTX treatment was discontinued in two patients due to these life-threatening adverse reactions. MTX-related neurotoxicity is commonly related with leukoencephalopathy, which is detected by magnetic resonance imaging (MRI) as white matter hyperintensities using T2-weighted and fluid-attenuated inversion recovery (FLAIR). Genomic DNA was isolated from each patient, and it was proved that having the CC genotype in rs2298383 (*ADORA2A*) was associated with an increased risk of leukoencephalopathy compared to patients who had the CT and TT genotypes. Although the clinical significance of asymptomatic leukoencephalopathy is unclear, decreased white matter volumes in ALL survivors were linked to attention and IQ deficits [27].

Ramalingam et al. conducted a study enrolling 115 children, aged 1 to 18 years with ALL. Using Sanger sequencing and PCR techniques, the authors evaluated the association of folate metabolism pathway genetic variants and chromosomal aberrations with treatment-related adverse events during MTX therapy. B-cell ALL patients receive 3 gm/m^2^ of MTX during the interim maintenance phase, while T-cell ALL patients receive 5 gm/m^2^. Co-trimoxazole is removed at least 6 days before the start of the maintenance phase with MTX. In this study, 115 children with acute lymphoblastic leukemia who were on maintenance therapy were enrolled; 9 of them relapsed and 9 died during treatment. Of the patients selected, 20 (17%) showed treatment-related adverse effects. In this study, *SLC19A1* (c.80G > A), Methylenetetrahydrofolate Reductase (c.677C > T; c.1298A > C), and *TYMS* (c.*450_*455del) genotypes were determined. Among the selected genotypes, *SLC19A1* 80G > A was significantly associated with a higher incidence of treatment-related adverse events, the other variants did not show a significant correlation. The monitoring of polymorphisms in selected candidate genes for the MTX metabolic pathway has the potential to help determine individualized dosing and to help moderate or reduce adverse events during chemotherapy [28].

Vora et al. conducted the UKALL2003 trial (United Kingdom National Randomised Trial For Children and Young Adults with Acute Lymphoblastic Leukaemia and Lymphoma 2003) and enrolled the biggest so far, a group of 3113 patients aged 1–24 years with ALL. There were 300 serious neurotoxicity adverse events report in 254 patients (8.2% of total trial participants), including 159 cases of encephalopathy, 86 cases of seizures, and 9 other cases. These individuals were compared to 2837 healthy controls who had no symptoms of neurotoxicity issues. Between the two groups, there was no significant difference in 5-year event-free survival, overall survival, or relapse risk. Treatment intensity, age, female sex, CNS status, T-cell immunophenotype, and white cell count were all found to have a significant relationship with neurotoxicity events. In the UKALL 2003 trial, most cases of neurotoxicity were reported due to encephalopathy associated with intrathecal methotrexate administration. The typical presentation is a focal neurological deficit, rapid personality changes, or loss of consciousness occurring within 1–21 days (mean 3 days) of exposure to intrathecal MTX. Full recovery was observed in most cases occurring within 48 h of the episode. However, in three cases methotrexate encephalopathy played a major role in the patient’s death. Although episodes occurred during all cycles of treatment, over half occurred during the consolidation or intensification phase. Unfortunately, this raises the possibility of interaction with cytarabine, which necessitated the establishment of recommendations for the management of encephalopathy during intrathecal MTX in the UKALL 2003 trial. No further intrathecal MTX should be administered while the patient is also receiving cytarabine (including in future courses containing cytarabine). These patients should be re-exposed, and if no recurrence occurs, missed doses should be administered during interim maintenance or maintenance. In the event of recurrence, a change to intrathecal cytarabine + hydrocortisone is needed [29].

Bhojwani et al. presented a prospective study of the significant clinical neurotoxicity of MTX. A total of 369 children with ALL participated in this study. The study included five high-dose MTX courses and 13 to 25 triplicate intravenous doses. During MTX treatment, 14 (3.8%) patients experienced clinical subacute neurotoxic episodes. Seizures were reported in seven patients, stroke-like symptoms in six patients, and ataxia in one patient. Twelve of thirteen patients who received I.V. and/or high-dose MTX had no recurrence of neurotoxicity. At least one screening MRI scan showed signs of leukoencephalopathy in 86 (23.3%) patients, including 73 (20.6%) asymptomatic patients and 13 (92.9%) out of 14 patients with clinical neurotoxicity. After treatment, 69% of those who developed leukoencephalopathy continued to have abnormal MRI findings. Aminophylline, which acts by competitive inhibition of adenosine, is a candidate for secondary prevention of MTX-related neurotoxicity, but the effectiveness of this therapy approach is unclear. In this study, one patient with recurrent MTX-related symptoms was given prophylactic aminophylline. After the first MTX-related neurotoxicity incident, the most common decision made by clinicians is to stop giving intrathecal MTX and only administer hydrocortisone and cytarabine. This technique, according to Bhojwani et al., is unnecessary because patients in their trial received 1 to 20 extra doses of intrathecal MTX without recurrence of neurotoxicity [30].

### 2.2. Vincristine

Vincristine is an anticancer drug that is widely used and effective in hemato-oncology. The incidence of neurotoxic consequences of using vincristine is unknown due to the variability of research groups [31]. VCR is metabolized significantly more efficiently by *CYP3A5* than by *CYP3A4* in in vitro tests. Patients with preB ALL who expressed *CYP3A5* showed less vincristine-induced peripheral neuropathy (VIPN), generated more M1, and had lower metabolic rates than those who did not express *CYP3A5* [32]. The CEP72 gene genetic variations have a lot of therapeutic potential [33]. Another genetic variant possibly related to the vincristine response is lower *NHP2L1* mRNA expression in leukemia cells derived from children with recently diagnosed ALL, which was substantially related to enhanced sensitivity to VCR in B- and T-cell leukemia [34].

The most serious side effect of VCRs is neurotoxicity, especially VIPN, which is divided into sensory, motor, and autonomic neuropathy, each of which causes a wide range of symptoms. Due to its varied clinical manifestations, the diagnosis of VIPN is difficult to establish in clinical practice. Sensory neuropathy is characterized by sensory nerve injury, which manifests as symmetry sensory/tactile impairment, numbness, and tingling in the hands and feet [35]. Peripheral sensory numbness, paresthesia, decreased balance, tendon weakening, and gait abnormalities are common symptoms of motor neuropathy [36]. Dysuria, erectile hypotension, sexual dysfunction, and paralytic ileus are all symptoms of autonomic neuropathy. VCR can also induce cranial neuropathy, which can lead to visual and hearing problems, as well as blindness and deafness [37]. VCR causes reversible mitotic arrest at low doses with little influence on spindle microtubule structure or polymerization. Higher VCR doses and long-term VCR exposure are associated with microtubule depolymerization-induced cytotoxicity. Furthermore, VCR obstructs tumor blood flow, resulting in tumor necrosis [38].

Adil et al. conducted a quasi-experimental study at the Multan Children’s Hospital and Children’s Health Institute in the period from January 2020 to October 2020. A total of 150 children aged 1 to 12 years old with ALL and Wilms’ tumor were included in the study, with 140 of them having ALL and passing the protocol chemotherapy with at least four consecutive weekly VCR injections. The study excluded children with any other neurological condition, CNS leukemia, prior chemotherapy, or relapse. Ptosis was found in 15 (10%) of the individuals. Jaw pain was reported in 12% of patients, hoarseness in 15% of patients, and constipation in 60% of patients. The rate of neurotoxic side effects of VCR is the same in both sexes. Children older than five years old were more likely to experience side effects. In these studies, the patients’ neurological indications and symptoms ranged from mild to moderate [39].

In 321 patients for whom DNA was accessible, a study by Barthelemy Diouf et al. investigated whole-genome single nucleotide polymorphism (SNP) and VIPN. A total of 222 patients were included in the St. Jude Children’s Research Hospital Total XIIIB protocol from 1994 to 1998, with toxic effects observed until January 2001, and 99 patients included in the Pediatric Oncology Group (COG) protocol AALL0433 from 2007 to 2010, with toxic effects observed until May 2011. During continuation therapy, 28.8% of patients in the St. Jude cohort and 22.2% of patients in the COG cohort had grade 2 to grade 4 VIPN. Patients with the high-risk *CEP72* genotype (TT at rs924607) had a greater rate of grade 2 to grade 4 neuropathy than those with the *CEP72* CC or CT genotypes, with 28 of 50 (56%) developing at least one episode [40].

Tay et al. used the clinical Total Neuropathy Score (cTNS) and nerve conduction investigations to assess VIPN in 101 ALL survivors aged 4–18 years who had completed chemotherapy for 2 years or more. Atypical cTNS results were found in 26.7% of respondents, whom all had benign neuropathy with a score of 1 to 4. Electrophysiological neuropathy affected 68.3% of patients, and of these 10.2% had only CNS sensorimotor disorder, 71.0% had both CNS and CNS sensorimotor disorder, and 18.8% had only CNS sensorimotor disorder. However, only 15.8% participants fulfilled the VIPN criteria for both cTNS and electrophysiological CNS abnormalities. There were no significant risk factors associated with abnormal TNS scores, and only the dose of VCR chemotherapy was associated with both electrophysiological neuropathy and VIPN; subjects with a medium/high risk of stratification had a higher risk of developing electrophysiological neuropathy (75.4 vs. 24.6%) and VIPN (67.3 vs. 32.7%) than those with a standard-risk group [41].

There is currently no gold standard for assessing VIPN, and most of the methods that are tested have limited effectiveness in young children, making it difficult to correctly quantify VIPN in this patient group. Electrodiagnostic testing is a possible distinct way for diagnosing VPN and elucidating the possible pathogenesis of peripheral nerve injury. This approach is more invasive and unpleasant for patients, making it unsuitable for routine evaluation of VIPN in children. However, VCR neuropathy is often reversible; nevertheless, it takes time to improve and can take months.

### 2.3. L-Asparaginase

Asparaginase breaks down L-asparagine to L-aspartic acid and ammonia, which results in decreased protein synthesis and apoptosis of leukemic cells that are unable to synthesize L-asparagine [42]. The enzyme is isolated from the bacteria Erwinia chrysanthemi or Escherichia coli, but the drug varies in pharmacokinetics and toxicity depending on which bacterium it is derived from [43]. Some studies have shown that up to 33% of children have CNS abnormalities during L-ASP treatment, but on the other hand there are some studies that have not detected any of such symptoms [44,45].

Preclinical studies have demonstrated that the mechanism of neurotoxicity of L-ASP results from the cleavage of aspartate and glutamine to aspartic acid, glutamic acid, and increased levels of ammonia [46]. Excessive stimulation of the NMDA (N-methyl-D-aspartate) receptor can cause cell death in CNS neurons, resulting in a large intracellular calcium influx and apoptosis. Increased concentrations of glutamate in cerebrospinal fluid in rats produced changes in T2-weighted CNS imaging comparable to those of patients treated with kainic acid, a glutamate analogue. [47] In addition, comparable changes were observed in some patients after an episode of prolonged seizure activity caused by cytotoxic edema, demyelination, or transependymal CSF resorption, which all can happen during L-ASP treatment [48].

The incidence of hypersensitivity and toxicity is the greatest during the consolidation and reinduction phases of therapy; however, the risk is even increased in patients who had prior exposure to asparaginase [49]. However, the diagnosis of neurotoxicity remains difficult due to the non-specific symptoms, which could be the result of exposure to a wide variety of chemotherapeutics during leukemia treatment.

CNS neurotoxicity caused by L-ASP might cause cerebral hemorrhage, thrombosis, parenchymal edema, and hyperammonemia [50]. The most common complication is CNS thrombosis occurring in 3–15% of cases, and the incidence of cerebral hemorrhage is estimated to be about 2% [51]. The most life threatening is toxic leukoencephalopathy caused by white matter damage. It manifests itself by personality and memory impairment in mild cases to coma and brain death in severe cases.

Kieslich et al. report five children aged between 8 and 14 years with neurologic complications during the first three weeks of L-ASP therapy due to ALL treatment. Four patients were treated with the ALL-BFM-95 protocol, whereas one patient was treated with the MCP-841 protocol. Three of the patients had venous thrombosis, one had a parenchymal hemorrhage, and one had a strange encephalopathy with extensive cortical and subcortical abnormalities that suggested a neurotoxic reaction. Furthermore, all the patients experienced a headache, three of them had additional seizures, and one of them had status epilepticus; all the patients survived complications. One child died later in the treatment as a result of a systemic disease that was resistant to treatment. Kieslich et al. emphasize that during L-ASP treatment, that even with mild neurologic symptoms, early imaging studies are mandatory and suggest that once a diagnosis of neurotoxicity is made, L-ASP should be discontinued [52].

Hyperammonemia, caused by catabolism of asparagine by L-ASP, is leading to increased ammonia levels. Too much ammonia in the brain is toxic and causes general CNS depression. The incidence of clinically significant hyperammonemia in children is unclear, but in adults, it may be around 10%, and the main symptoms reported include fatigue. Treatment of hyperammonemia is analogical to what is proposed in urea cycle disorders, aiming to decrease glutamine concentration and consequently hyperammonemia. The medical management included a decrease in protein intake and lactulose, which was given to reduce intestinal ammonia production. Moreover, Sodium Benzoate reacts with glycine and Sodium Phenylbutyrate reacts with glutamine, and this treatment (monitored on ammonia and glutamic acid levels) appeared to be effective as long as asparagine is decreased due to asparaginase treatment [53].

The incidence of thrombotic complications associated with L-ASP is clearly age dependent. In children, the overall incidence of L-ASP-related thrombosis varies from 2.4% to 11.5% [54]. In the study by Grace et al., age was the only significant predictor of asparaginase-related thrombosis, and the incidence of venous thromboembolic events was 5% among pediatric patients, 34% in adult patients, and 42% in adults aged 30 years [55].

A meta-analysis conducted by Caruso et al. to evaluate thrombotic complications in pediatric ALL included 17 studies involving 1752 patients. The incidence of thrombotic complications in all patients was 5.2%. The authors reported that most events (53.8%) occurred in the CNS, and 28.6% of all events were classified as central venous thrombosis. Patients who received asparaginase for more than 9 days were more likely to have thrombosis, but there was no significant difference in the incidence of thrombotic events in patients who received asparaginase 2–3 times per week compared with patients who received the drug continuously [56].

### 2.4. Nelarabine

Nelarabine (Ara-G) is a novel purine analog that is used to treat T-cell acute lymphoblastic leukemia (T-ALL) and T-cell lymphoblastic lymphoma (T-LBL) patients whose illness has not responded to or relapsed after at least two chemotherapy regimens [57]. Ara-G has not been well tested in those with newly diagnosed T-ALL. For both juvenile and adult patients, neurologic toxicity was dosage limiting. The addition of nelarabine to ABFM (augmented Berlin–Frankfurt–Muenster) therapy enhanced DFS without increasing toxicity in children and young adults with newly diagnosed T-ALL [58]. Ara-G is a DNA-terminating nucleoside prodrug for araguanosine, which is converted into ara-G triphosphate (ara-GTP) and accumulates preferentially in T lymphoblasts due to its slow breakdown kinetics. The amount of intracellular ara-GTP accumulated determines the efficacy of nelarabine. The severity and frequency of neurological adverse events varied significantly across all cohorts and treatment regimens reported to date. Previous treatment with neurotoxic agents such as VCR, high-dose cytarabine, high-dose MTX, intrathecal chemotherapy, and/or cranial irradiation may impact Ara-G-related neurological side effects.

Dunsmore et. al., in her original report about a phase III randomized clinical trial testing Ara-G, examined 1562 evaluable patients with T-ALL: newly diagnosed, untreated (except corticosteroids), and aged 1–31 years. This randomization did not include low-risk participants. Adverse events were graded using CTCAE 4.0 (Common Terminology Criteria for Adverse Events version 4). Overall toxicity and neurotoxicity were acceptable and similar between all four randomized arms. The rate of peripheral neurotoxicity of grade ≥ 3 was 15%. In the arms with and without Ara-G, there was no significant difference in the frequencies of grade 3 central neurotoxicity (encephalopathy, leukoencephalopathy, CNS necrosis, reversible posterior leukoencephalopathy syndrome, Guillain–Barre syndrome, cerebral edema, and pyramidal tract syndrome) (3.4% vs. 2.1%). This study, along with COG AALL00P2, found that Ara-G was not linked to an elevated risk of neurotoxicity in newly diagnosed patients, although there was the presence of uncommon serious neurotoxicities (3 of 366 patients: 0.8%) [59].

Between December 2011 and September 2018, Kumamoto and colleagues recruited patients with first-relapsed T-ALL, older than 1 year and younger than 18 (*n* = 6). There was no grade 3–4 non-hematological toxicity, including neurotoxicity, except for febrile neutropenia (three cycles) [60].

Kuhlen et al. analyzed 52 patients with relapse or refractory (R/R) T-ALL/T-LBL aged ≤19 years who were treated with Ara-G alone (*n* = 25) or in combination with cyclophosphamide (5 × 440 mg/m^2^) and etoposide (5 × 100 mg/m^2^) (*n* = 27) in a retrospective study. Twelve patients (23.1%) suffered neurotoxic adverse events of any grade, with seven (13.5%) developing neurotoxicity grade 3 as defined by CTCAE 4.03. Peripheral motor neuropathy (19.2%), peripheral sensory neuropathy (11.5%), and seizures (9.6%) were the most common neurologic adverse event. After 1–2 cycles of combination therapy, three patients died of a neurologic adverse event. Neurotoxic patients were much older than individuals who did not have it. Univariate and multivariate analyses identified older age as a risk factor. Only 2 of the 12 (16.7%) patients who suffered neurotoxicity survived. After nelarabine therapy, both patients were in complete response at the latest follow-up, one at 5 months and the other at 51 months. In terms of results and neurologic adverse event, there were no differences between mono and combination treatment [61].

## 3. Neurotoxicity of Immunotherapy

Several of the newly discovered molecular alterations have led to the development of approaches that focus on the dysregulation of cellular pathways [5]. Despite tremendous advances in the treatment of ALL, no drug has shown more promise in improving survival outcomes than immunotherapeutic approaches. However, increasing evidence suggests that immunotherapy also induces serious and complex neurologically related adverse events and may even lead to related deaths, raising concern among clinicians for their more widespread use [53]. Nevertheless, neurotoxicity caused by immunotherapy should not be underestimated. Early and accurate diagnosis of neurotoxicity increases the effectiveness of treatment, but its mechanism of neurotoxicity has not yet been fully elucidated [21]. For this reason, treatment of neurotoxicity is limited only to the clinician’s own experience.

### 3.1. Blinatumomab

Dual-specific T-cell-binding antibodies (BiTEs) are made up of two antibodies with varying antigen-binding domains joined by a non-immunogenic binding peptide. One of the most clinically advanced BiTEs is blinatumomab, generated from a B-lineage-specific mouse monoclonal antibody. Blinatumomab is made up of two arms: one for attaching CD3-expressing T-cells and another for attaching CD19+ B-cells. Blinatumomab is approved for the treatment of precursor B-ALL in first or second complete remission with minimal residual disease (MRD) greater than or equal to 0.1% in adults and children [62].

There are reports that overall neurologic toxicities with blinatumomab treatment occur in 15–50% of patients. Neurologic adverse events can be severe, life threatening, or fatal, and grade 3 or higher neuropsychiatric toxicity has occurred in approximately 15% of patients [63]. The mechanism of blinatumomab-induced neurotoxicity is still unknown. However, CNS events are presumed to be caused by an inflammatory irritation of the myoendothelium by blinatumomab-activated T-cells, which locally release neurotoxic cytokines and chemokines locally on their way into the CNS [64]. Adverse neurologic events may include tremor, slurred speech, loss of vibratory sensation, dizziness, confusion, encephalopathy, and seizure, which are the most common manifestations of neurotoxicity. In adult patients, symptoms of neurotoxicity began after an average of 9 days, but pediatric patients may manifest earlier [65].

Queudeville et al. observed response to blinatumomab in 34% of pediatric patients with R/R ALL [66]. However, despite promising results, side effects are still common. The incidence of blinatumomab-associated neurotoxicity was 54% in adult patients with R/R ALL and 24% in a similar pediatric cohort [67].

Locatelli et al. conducted a randomized, phase III clinical trial on 108 children to evaluate event-free survival in children with high-risk first-relapse B-ALL after a third consolidation course with blinatumomab vs. consolidation chemotherapy before HSCT. The incidence of events in the blinatumomab vs. consolidation chemotherapy groups was 31% vs. 57% after a median of 22.4 months of follow-up. Deaths occurred in 16 (29.6%) patients in the consolidation chemotherapy group and 8 (14.8%) patients in the blinatumomab group. In the blinatumomab vs. consolidation chemotherapy group, the incidence of a serious adverse event was 24.1% vs. 43.1%. In the blinatumomab group, the most common serious adverse events were neurologic symptoms and seizures (each 3.7%) compared to febrile neutropenia (17.6%) in the consolidation chemotherapy group. Based on the findings of this trial, blinatumomab medication may be a helpful consolidation treatment for this patient population, as it appears to be more successful than conventional chemotherapy before transplant [68].

Beneduce et al. conducted a multicenter retrospective study where they evaluated blinatumomab in children and adolescents with R/R B-Cell precursor ALL. The aim of their study was to retrospectively assess the efficacy and safety of blinatumomab in 39 children treated in 7 AIEOP (Associazione Italiana di Ematologia e Oncologia Pediatrica) centers. Blinatumomab proved to be very effective, and treatment was well tolerated. The pediatric dosage of stepwise escalation from 5 to 15 μg/m^2^/day in the first course and subsequently 15 μg/m^2^/day in future courses was given to the majority of children (21, 53%). Four patients were treated with the adult schedule, three with escalation from 9 to 28 μg/day, and one with 28 μg/day from day one; fourteen patients (36%) received the full pediatric dosage from day one. Total number of adverse events were 46, of which 27 of them were grade ≤ 2. A total of 14 (36%) patients experienced neurologic adverse events: 3 patients had seizures, 2 patients displayed tremors, and 2 displayed peripheral neuropathies. One patient reported aphasia and dysarthria associated with tremor and dysmetria, and two patients had multiple neurological adverse events. However, no associated toxic deaths were reported [69].

Stein et al. conducted a trial to evaluate the neurologic adverse event on 189 adult R/R ALL patients treated with blinatumomab. A total of 98 patients (52%) experienced neurologic adverse events, most frequently dizziness, tremor, confusional state, and encephalopathy. Neurologic adverse events were most common in cycle 1 (median onset, 9 days) and were usually grade 1 to grade 2. Infusion interruptions or dexamethasone administration were used to treat grade 3 neurologic adverse events (13–17%) and serious neurologic adverse events (16–19%). The incidence of neurotoxicity increased with increasing blinatumomab concentration at a given dose; however, exposure appeared unrelated to the severity of neurologic adverse events. If neurologic events were grade 3 or severe neurologic events occurred, the blinatumomab infusion was stopped immediately. If neurologic events returned to grade ≤ 1, blinatumomab could be restarted at 9 μg/day (no dose escalation was allowed) after a 2 week interval without treatment. Before restarting blinatumomab, patients were premedicated with dexamethasone (and prophylactic anticonvulsant, if used) [67].

There are no specific guidelines or consensus established for managing the neurologic adverse events during blinatumomab infusion, and the methods being adopted currently are broadly based on clinical experience. Data on the use of blinatumomab in pediatrics are still lacking, and results from studies in adults cannot simply be transferred to pediatrics.

For patients with bone marrow blasts of more than 50%, peripheral blood blasts of 15,000 cells per μL or higher, or elevated lactate dehydrogenase suggesting rapidly progressing disease, some scientists required prephase treatment with dexamethasone 10–24 mg/m^2^ per day (for up to 5 days) to reduce the incidence of severe cytokine release syndrome. However, after the completion of the studies, it was found that pre-treatment with dexamethasone had no effect on the response [63].

### 3.2. Inotuzumab Ozogamicin

Inotuzumab ozogamicin (InO) is an antibody–drug conjugate composed of a monoclonal CD22-directed antibody linked to calicheamicin, a potent cytotoxic antitumor antibiotic that induces apoptosis by breaking double-strand DNA [70]. In 2017, the FDA approved InO for treating adults with CD22-positive R/R B-cell precursor ALL [71]. Unfortunately, because of the small number of randomized trials of InO in children, it is difficult to estimate the incidence of neurotoxicity. There are no studies on the mechanism of neurotoxicity either [72].

A recent report from the COG study aimed to prospectively determine safety and efficacy of InO in pediatric and adolescent patients with R/R B-ALL. Patients in the InO arm received 1.8 mg/m^2^ intravenously each cycle, for a maximum of six cycles. Patients in the chemotherapy arm received cytarabine with mitoxantrone, FLAG (fludarabine, cytarabine, and granulocyte colony-stimulating factor [GCSF]), or high-dose cytarabine, as determined by the investigator. Among 48 patients, 19 patients achieved a complete response (CR), and 9 patients achieved a CR with incomplete cell count recovery (CRi) after cycle 1 of InO. However, in all studies using InO, hepatotoxicity, particularly sinusoidal obstruction syndrome (SOS), is one of the most life-threatening adverse events due to mortality rates exceeding 80% in patients who develop multiple organ failure. Side effects described in InO drug information related to CNS include only headache, which means that InO appears to be safe and well tolerated in terms of neurotoxicity [73].

Kantarjian et al. conducted a trial on 326 patients treated for ALL which were randomly assigned to receive either InO or standard intensive chemotherapy. The percentage of patients who had serious adverse events was similar in the InO group and the standard-therapy group: 48% and 46%, respectively. However, in neither group was death associated with neurotoxicity [74].

### 3.3. CAR T-Cell Therapy

**Figure 1:**
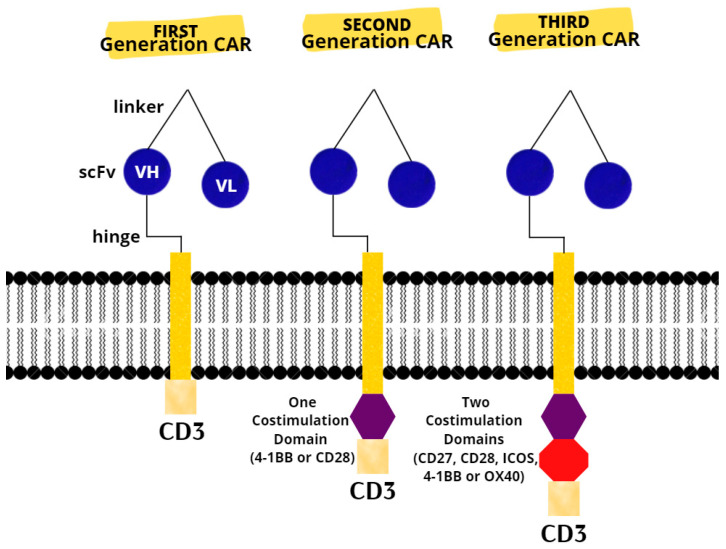
Chimeric antigen receptors. Next-generation CARs have additional modifications to their intracellular stimulatory domains. CD3, cluster of differentiation 3; ICOS, Inducible T-cell costimulator; scFv, single-chain fragment variable; VH, heavy chain variable gene segment; VL, variable region.

The last resort for acute or refractory ALL in patients up to 25 years of age are two CAR T products (tisagenlecleucel and axicabatagene ciloleucel) that have recently been approved for treatment in the United States and Europe [75]. There are three generations of CARs (Figure 1). A CAR T-cell’s basic structure usually consists of a tumor-targeting domain derived from a monoclonal antibody linked to a CD3 zeta chain that serves as an intracellular signaling domain. A co-stimulatory endodomain, either 4-1BB or CD28, is also present in second-generation CARs [76]. A third-generation CAR T-cell’s purpose is to increase T-cell proliferation and persistence by combining signaling domains such as 4–1BB, OX40 (CD134), inducible T-cell costimulator (ICOS), and CD27, to boost the cytotoxic effect [77].

**Figure 2:**
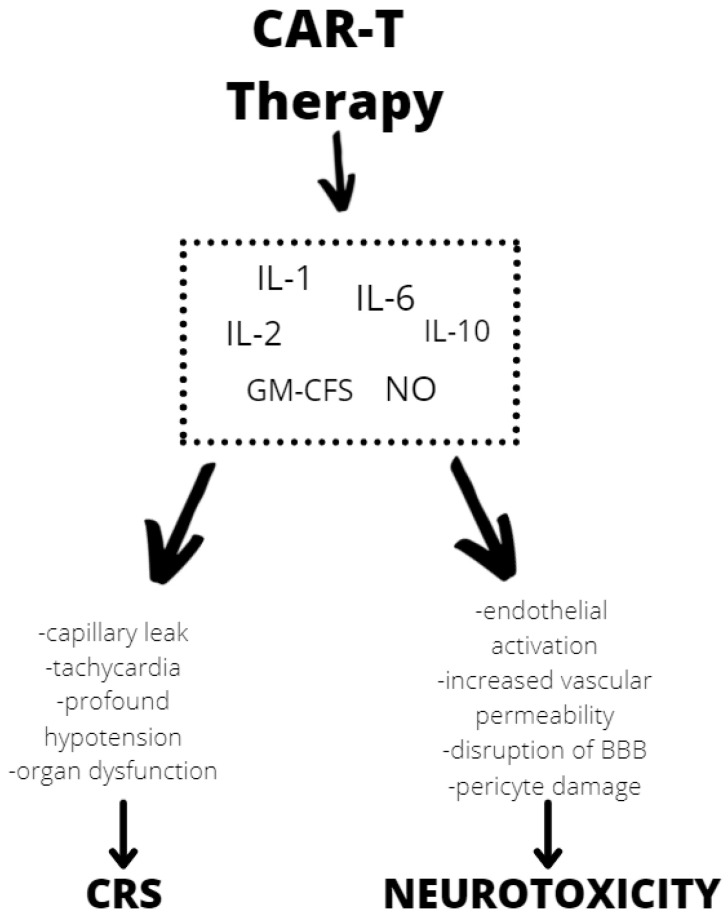
Mechanisms of neurotoxicity and cytokine release syndrome (CRS) caused by CAR T therapy. BBB—blood–brain barrier, GM-CFS—granulocyte-macrophage colony stimulating factor, NO—nitric oxide.

One of the most severe complications of CAR T-cell is neurotoxicity. Neurologic adverse effects were observed in 40–44% of children and young adults in studies using CAR constructs with a 4-1BB domain [78,79]. Another study using CD19 CAR with a CD28 costimulatory domain detected neurotoxicity in 30% (5% severe) of children and young adults. CAR T-cell therapy has been linked to specific toxicity of the CRS and neurological toxicity [80]. Although the exact mechanism of neurotoxicity is unknown, evidence shows endothelial activation and increased blood–brain barrier permeability, which results in a high cytokine concentration in the cerebrospinal fluid. These cause more endothelial cell and pericyte activation, which, if severe, might result in cerebral edema or other CAR T neurotoxicity manifestations (Figure 2). In contrast to the previously assumed mediators derived from T lymphocytes, after CAR T infusion we observed a significant increase in the level of cytokines, including granulocyte-macrophage colony growth factor (GM-CSF), IL-10, IL-6, and IL-1 and IL-6 generated from host macrophages that have been shown to mediate neurotoxicity. Depending on the kinetics of T-cell proliferation, CRS with CAR T might develop shortly after infusion or be a delayed response that occurs days or weeks later [81].

**Figure 3:**
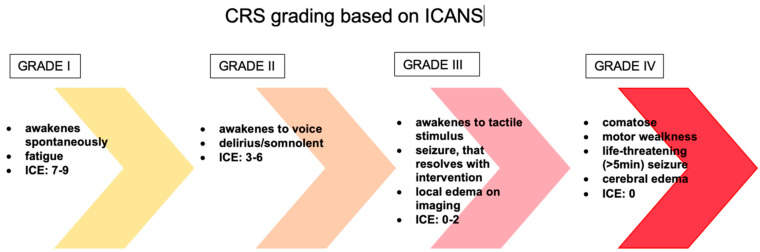
The management of ICANS is based on a grading system. CRS—cytokine release syndrome, ICANS—immune effector cell-associated neurotoxicity syndrome, ICE—Immune Effector Cell-Associated Encephalopathy score.

The evaluation and classification of these toxicities vary greatly between clinical trials and institutions, making it difficult to compare the safety of different medicines and to create effective care methods for these toxicities. The first patients treated with CD19 CAR T-cells experienced similar toxicities to those observed with the immunomodulatory drug theralizumab (TGN1412), including aggressive behavior, rigidity, fever, poor concentration, and psychosis [82]. Supraphysiologic cytokine increase was found to be responsible for the great majority of symptoms in the first pediatric ALL patient treated with CAR T-cell therapy, implying that these toxicities were caused by CRS. Encephalopathy, agitation, aphasia, tremor, lethargy, delirium, difficulty concentrating, seizures, and in rare cases even cerebral edema are all symptoms of ICANS [83]. In addition, headache is a very common symptom that may or may not indicate neurotoxicity (Figure 3). Over the years, many scales have been developed, such as CTCAE (Common Terminology Criteria for Adverse Events) version 4.03, CTCAE version 5.0, Lee criteria, and Peen criteria et al., which redefined the scoring criteria for CRS. Many CAR T-cell groups have adopted the Lee criterion, in part because it was the first to link a specific grade to a suggested therapy protocol. As previously indicated, neurotoxicity is a common side effect of CAR T-cell and other T-cell-engaging therapies. Neurotoxicity associated with immune effector cells, unlike classic CRS symptoms, do not often respond to tocilizumab treatment. This fact is not surprising, given that when tocilizumab is administered I.V., large amounts of the drug do not accumulate in the CSF. Symptoms of ICANS can be more diverse than those of CRS. Many patients with neurotoxicity have a stereotypic evolution of a specific set of symptoms. The earliest manifestations of ICANS are mild difficulty with expressive speech (especially in naming objects), dysgraphia, impaired attention, tremor, apraxia, and mild lethargy [84]. Early detection of marker changes such as peak C-reactive protein (CRP), ferritin on day 3 but not peak ferritin, and fever can have a huge impact on the prognosis of patients who develop neurotoxicity. In people at risk of neurotoxicity, the number of cytokines such as IL-6, IL-10, granulocyte-macrophage colony stimulating factor, IL-15, IL-2, and the TNF receptor should also be determined. However, it is important to realize that none of these markers are unique to chemotherapy or immunotherapy or CRS induced neurotoxicity. Only the combination of several ingredients gives a picture of whether a given patient is at risk of neurotoxicity. High-dose methylprednisolone is typically used in the most severe cases of CAR T-cell-related neurotoxicity. Most neurotoxic patients also have CRS, which can be treated with tocilizumab, an IL-6 receptor antibody, and/or corticosteroids to suppress T-cell activation, but the effect of these medications on neurotoxicity is uncertain [85].

Shah et al., who developed a novel CD22-targeted/4-1BB CAR T-cell and tested it in a phase I dose–escalation trial in children and young adults with R/R CD22 hematologic malignancies, described a dose-dependent antileukemic response in CD19-negative/dim or CD191 relapsed ALL patients with an acceptable toxicity profile that included limited CRS, minimal neurotoxicity, and an effective signal that was not altered by earlier CD19 targeting. All patients were given dose levels based on the number of transduced CAR T-cells per kilogram; the first dose level was 3 × 10^5^/kg, the second 1 × 10^6^/kg, and the third 3 × 10^6^/kg. They also received fludarabine 25 mg/m^2^/d on days 24, 23, and 22, and cyclophosphamide 900 mg/m^2^ on day 22 with CD22 CAR T-cell infusion. This was a group of 64 patients, of which 58 received infusion and were evaluated for toxicity. Fifty (86.2%) of fifty-eight participants developed CRS which was grade 1–grade 2 in 45 days (90%). Two CRS symptom grade 5 events occurred at the second dose level, and one in the setting of Gram-negative sepsis and multiorgan dysfunction. Neurotoxicity was typically minor in the first 22 patients, with no seizures, encephalopathy, or more severe symptoms of toxicity. Except for 1 patient who experienced grade 4 cerebral bleeding, 19 (32.8%) of the 58 participants had one or more recorded neurologic manifestation, all of which were grade 1 and grade 2 toxicity intracranial hemorrhage (ICH). A total of 57 people who received an infusion were evaluated for response; one person with grade 5 CLS died before the disease was re-staged [86]. Gardner et al. enrolled 45 patients with relapsed or refractory CD19+ ALL ages 1–27 years old. The study’s principal goal was to assess feasibility and toxicity. CRS and neurotoxicity were the most frequent side effects of CAR T-cells. CRS was found in 93% (40 of 43) of patients, with a rate of 23% of patients having severe CRS (requiring pressors, inotropes, or respiratory failure). The overall neurotoxicity was found in 49% of cases (21 out of 43); severe neurotoxicity (any degree of seizure or grade 3 to grade 4 neurotoxicity exclusive of headache) was found in 21% of cases (9 of 43) [87].

Santomasso et al. conducted a trail to evaluate the clinical and biological correlation of neurotoxicity associated with CAR T-cell therapy in patients with B-cell ALL: 62.3% of patients experienced neurotoxicity of any grade 28 days after CAR T-cell injection, while brain edema or catastrophic neurotoxicity occurred in 53 patients. Eleven (20.8%) patients experienced moderate neurological symptoms, twenty-two (41.5%) patients experienced severe neurotoxicity more than grade 3, nineteen (35.8%) patients experienced grade 3, and three (5.7%) patients experienced grade 4 neurologic events. Mild encephalopathy or delirium, headache, and tremor were the most observed neurologic symptoms among the 11 patients who had mild neurotoxicity. Mild somnolence, disorientation, decreased attention, and trouble naming were common symptoms of severe neurotoxicity before escalating to global aphasia, myoclonus, depressed level of consciousness, dementia, and seizures. Neurotoxicity lasted an average of 11 days (range: 2–92 days) in patients who had severe symptoms The presence and severity of neurotoxicity were found to have a strong relationship with CRS [88].

## 4. Conclusions

Despite several studies, many questions remain unresolved. In chemotherapy, the key clinical question for clinicians should be whether re-exposure to chemotherapy drugs following a major neurological event is appropriate and how to use genetic testing results that indicate a higher potential for neurotoxicity. CAR T-cell therapy and immunotherapy have changed the treatment landscape of ALL, in particular R/R ALL, in a relatively short period of time. However, if the use of chemotherapy combined with innovative drugs becomes more widespread, clinicians will need to be able to recognize and treat the associated toxicity sooner. Understanding the early neurotoxic variables should perhaps reduce the incidence of side effects. Corticosteroids, IL-6 targeted therapies, and adjuvants are often used to treat patients with neurotoxicity, but high-quality evidence for efficacy is lacking, and most treatments for neurotoxicity are based on the clinician’s own experience. There is a need for multicenter studies that would provide clear guidelines for the management of neurotoxicity that would simplify the daily management of patients.

## Figures and Tables

**Table 1:** Treatments used in ALL and associated neurotoxicity.

Phase of Treatment	Drugs	Toxicity-Related Gene	Mechanism of Neurotoxicity	Neurotoxicity	References
Induction	Vincristine	*ABCC11* ^1^, *ABCC2* ^2^, *ABCC4* ^3^, *ABCC5* ^4^, *ABCB1* ^5^, *ABCC10* ^6^, *CEP72* ^7^, *SLC5A7* ^8^, *TUBB1* ^9^, *TUBB2A* ^10^, *TUBB2B* ^11^, *TUBB3* ^12^, *TUBB4A* ^13^, *MAP4* ^14^, *CYP3A4* ^15^, *CYP2C8* ^16^, *CYP3A5* ^17^, *CEP72* ^18^	Interferes with the assembly of microtubule structures leading to cell apoptosis. It affects the peripheral nerves but can also contribute to dysfunction of the cranial nerves and autonomic nervous system.	Peripheral neuropathy, sensory neuropathy: symmetry sensory/tactile impairment, numbness, and tingling in the hands and feet, paresthesia, decreased balance, tendon weakening, visual and hearing problems.	[8,9]
	L-asparaginase	*ZBTB1* ^19^, *GRIA1* ^20^, *HLA-DRB1* ^21^	L-asparaginase produces three neurotoxic agents: ammonia, L-aspartic acid, and glutamic acid. These two amino acids can induce cell death in CNS neurons by excessive stimulation through NMDA (N-methyl-D-aspartate) receptor, leading to a major intracellular calcium influx and apoptosis.	Myelosuppression, encephalopathy, hepatic toxicity.	[10,11,12]
Consolidation	Methotrexate (intravenous infusion and intrathecally)	*DHFR19bp* ^22^, *MTHFR 677C > T* ^23^, *MTHFR 677TT* ^24^, *SLC19A1* ^25^, *TYMS* ^26^, *ADORA2A* ^27^	Methotrexate is an antimetabolite that inhibits Dihydrofolate Reductase and thus tetrahydrofolate formation. This affects the synthesis of macromolecules such as myelin, and reversible leukoencephalopathy has been suggested to be secondary to impaired myelin turnover. Dihydrofolate Reductase inhibition leads to lack of folate and cobalamin, and increase in homocysteine, which is toxic to vascular endothelium may cause seizures and vascular disease. Dihydrofolate Reductase inhibition results in decreased levels of S-adenosylmethionine, which in turn plays a role in maintaining the myelin sheath, and this deficiency may lead to demyelination after intrathecal methotrexate administration.	Transverse myelopathy-symptoms include back pain with subsequent weakness, sensory loss and bladder or bowel incontinence, blurred vision, aphasia, anarthria, seizures, aphasia, mental status disorder, stroke-like episodes, delirium, leukoencephalopathy septic meningitis characterized by headache, neck stiffness, nausea, vomiting and potential fever and encephalopathy.	[13,14,15]
	Cytarabine	*DCK* ^28^, *NT5C2* ^29^, *CDA* ^30^, *RRM1* ^31^, *GIT1* ^32^, *NT5C 3* ^33^, *ENT1* ^34^, *SCL29A1* ^25^	Cytarabine exhibits preferential toxicity for CNS ^35^ progenitor cells and oligodendrocytes, compromises cell division in vitro, and causes cell death and reduced cell division in vivo.	Myelosuppression, neurotoxicity.	[16,17,18,19]
Maintenance	Methotrexate (orally)	Genes have been described above.	Mechanism has been described above.	Seizures, aphasia, mental status disorder, stroke-like episodes, delirium, leukoencephalopathy, cognitive dysfunction, personality changes.	[13,14,15,20]

^1^ ABCB11, ATP Binding Cassette Subfamily C Member 11; ^2^ ABCC2, ATP Binding Cassette Subfamily C Member 2; ^3^ ABCC4, ATP Binding Cassette Subfamily C Member 4; ^4^ ABCC5, ATP Binding Cassette Subfamily C Member 1; ^5^ ABCB1, ATP Binding Cassette Subfamily B Member 1; ^6^ ABCC10, ATP Binding Cassette Subfamily C Member 10; ^7^ CEP72, Centrosomal Protein 72; ^8^ SLC5A7, Solute Carrier Family 5 Member 7; ^9^ TUBB1, Tubulin Beta 1 Class VI; ^10^ TUBB2A, Tubulin Beta 2A Class IIa; ^11^ TUBB2B, Tubulin Beta 2B Class IIb; ^12^ TUBB3, Tubulin Beta 3 Class III; ^13^ TUBB4A, Tubulin Beta 4A Class Iva; ^14^ MAP4, Microtubule-Associated Protein 4; ^15^ CYP3A4, Cytochrome P450 Family 3 Subfamily A Member 4; ^16^ CYP2C8, Cytochrome P450 Family 2 Subfamily C Member 8; ^17^ CYP3A5, Cytochrome P450 Family 3 Subfamily A Member 5; ^18^ CEP72, Centrosomal Protein 72; ^19^ ZBTB1, Zinc Finger and BTB Domain Containing 1; ^20^ GRIA1, Glutamate Ionotropic Receptor AMPA Type Subunit 1; ^21^ HLA-DRB1, Major Histocompatibility Complex Class II, DR Beta1; ^22^ DHFR19bp, Dihydrofolate Reductase 19 bp polymorphism; ^23^ MTHFR 677C > T, Methylenetetrahydrofolate Reductase polymorphism; ^24^ MTHFR 677TT, Methylenetetrahydrofolate Reductase polymorphism; ^25^ SCL29A1, Solute Carrier Family 29, member 1; ^26^ TYMS, Thymidylate Synthetase; ^27^ ADORA2A, Adenosine A2a Receptor; ^28^ DCK, Deoxycytidine Kinase; ^29^ NT5C2, 5’-Nucleotidase, Cytosolic II; ^30^ CDA, Cytidine Deaminase; ^31^ RRM1, Ribonucleotide Reductase Catalytic Subunit M1; ^32^ GIT1, G Protein-Coupled Receptor Kinase Interacting ArfGAP 1; ^33^ NT5C3, 5’-Nucleotidase, Cytosolic IIIA; ^34^ ENT1, Equilibrative nucleoside transporter 1; ^35^ CNS, central nervous system.

## Data Availability

No new data were created or analyzed in this study. Data sharing is not applicable to this article.

## Abbreviations

ABCB1	ATP Binding Cassette Subfamily B Member 1
ABCB11	ATP Binding Cassette Subfamily C Member 11
ABCC10	ATP Binding Cassette Subfamily C Member 10
ABCC2	ATP Binding Cassette Subfamily C Member 2
ABCC4	ATP Binding Cassette Subfamily C Member 4
ABCC5	ATP Binding Cassette Subfamily C Member 1
ADHD	attention deficit hyperactivity disorder
ADORA2A	Adenosine A2A Receptor
AIEOP	Associazione Italiana di Ematologia e Oncologia Pediatrica
ALL	acute lymphoblastic leukemia
ALL-BFM-95	ALL-Berlin–Frankfurt–Münster
Ara-G	Nelarabine
ara-GTP	ara-G triphosphate
Arg27His	amino acid arginine to histidine at codon 27
BBB	blood–brain barrier
BiTEs	dual-specific T-cell-binding antibodies
CD3	cluster of differentiation 3
CDA	Cytidine Deaminase
CEP72	Centrosomal Protein 72
CNS	central nervous system
COG	Pediatric Oncology Group
CR	complete response
Cri	a complete response with incomplete cell count recovery
CRP	C-reactive protein
CRS	cytokine release syndrome
CSF	cerebrospinal fluid
CTCAE	Common Terminology Criteria for Adverse Events
cTNS	clinical Total Neuropathy Score
CYP2C8	Cytochrome P450 Family 2 Subfamily C Member 8
CYP3A4	Cytochrome P450 Family 3 Subfamily A Member 4
CYP3A5	Cytochrome P450 Family 3 Subfamily A Member 5
DCK	Deoxycytidine Kinase
DHFR19bp	Dihydrofolate Reductase 19 bp polymorphism
ENT1	Equilibrative nucleoside transporter 1
GIT1	G Protein-Coupled Receptor Kinase Interacting ArfGAP 1
GM-CSF	granulocyte-macrophage colony stimulating factor
GRIA1	Glutamate Ionotropic Receptor AMPA Type Subunit 1
HLA-DRB1	Major Histocompatibility Complex Class II, DR Beta1
HSCT	allogeneic hematopoietic stem cell transplant (HSCT)
I.V.	intravenous
ICANS	immune effector cell-associated neurotoxicity syndrome
ICE	Immune Effector Cell-Associated Encephalopathy score
ICH	intracranial hemorrhage
ICOS	Inducible T-cell costimulator
ID-MTX	intermediate-dose methotrexate
InO	Inotuzumab
L-ASP	L-asparaginase
MAP4	Microtubule-Associated Protein 4
MRD	minimal residual disease
MTHFR 677C>T	Methylenetetrahydrofolate Reductase polymorphism
MTHFR 677TT	Methylenetetrahydrofolate Reductase polymorphism
MTX	methotrexate
MTX-NT	neurotoxicity associated with methotrexate
NO	nitric oxide
NT5C2	5’-Nucleotidase, Cytosolic II
NT5C3, 5’	Nucleotidase, Cytosolic IIIA
R/R	relapsed or refractory
RRM1	Ribonucleotide Reductase Catalytic Subunit M1
scFv	single-chain fragment variable
SLC19A1	Solute Carrier Family 19 Member 1
SLC5A7	Solute Carrier Family 5 Member 7
SNPs	Single nucleotide polymorphisms
SOS	sinusoidal obstruction syndrome
T-ALL	T-cell acute lymphoblastic leukemia
T-LBL	T-cell lymphoblastic lymphoma
TIT	triple intrathecal therapy
TUBB1	Tubulin Beta 1 Class VI
TUBB2A	Tubulin Beta 2A Class IIa
TUBB2B	Tubulin Beta 2B Class IIb
TUBB3	Tubulin Beta 3 Class III
TUBB4A	Tubulin Beta 4A Class Iva
TYMS	Thymidylate Synthetase
UKALL2003 trial	United Kingdom National Randomised Trial For Children and Young Adults with Acute Lymphoblastic Leukaemia and Lymphoma 2003
VCR	vincristine
VH	heavy chain variable gene segment
VL	variable region
VIPN	vincristine-induced peripheral neuropathy
ZBTB1	Zinc Finger and BTB Domain Containing 1
